# A Dissipative Connector for CLT Buildings: Concept, Design and Testing

**DOI:** 10.3390/ma9030139

**Published:** 2016-02-26

**Authors:** Roberto Scotta, Luca Marchi, Davide Trutalli, Luca Pozza

**Affiliations:** Department of Civil, Environmental and Architectural Engineering, University of Padova, Via Marzolo 9, Padova 35131, Italy; roberto.scotta@dicea.unipd.it (R.S.); davide.trutalli@dicea.unipd.it (D.T.); luca.pozza@dicea.unipd.it (L.P.)

**Keywords:** CLT, X-Lam, dissipative connections, behavior factor, dissipative capacity, seismic response

## Abstract

This paper deals with the conception and characterization of an innovative connection for cross-laminated timber (CLT) panels. The connection is designed to provide an adequate level of dissipative capacity to CLT structures also when realized with large horizontal panels and therefore prone to fragile shear sliding failure. The connector, named X-bracket, has been theorized and designed by means of numerical parametric analyses. Furthermore, its cyclic behavior has been verified with experimental tests and compared to that of traditional connectors. Numerical simulations of cyclic tests of different CLT walls anchored to the foundation with X-brackets were also performed to assess their improved seismic performances. Finally, the analysis of the response of a 6 m × 3 m squat wall demonstrates that the developed connection provides good ductility and dissipation capacities also to shear walls realized with a single CLT panel.

## 1. Introduction

The seismic behavior of CLT buildings has been studied by numerous researchers from various countries. Quasi-static tests of shear-wall systems and shake-table tests of full-scale buildings [1,2,3,4,5] showed that CLT structures are characterized by high strength and stiffness when subjected to seismic actions. However, they might exhibit low ductility and dissipative capacity if not correctly designed to prevent brittle failure or if realized with large and continuous wall elements, without vertical joints (*i.e.*, if characterized by prevailing sliding behavior). In the building practice, the adoption of large panels with few joints allows the reduction of time and costs for on-site assembling. However, the use of narrow panels allows one to optimize the use of the material and to reduce the weight and dimensions to be lifted and transported. 

The current version of European seismic code Eurocode 8 [6] does not consider explicitly CLT as a structural system, and the closest definition is “glued wall panels with glued diaphragms”. Therefore, CLT is classified as a low-ductility system, and a behavior factor q_0_ = 2 is suggested for the design of CLT buildings, regardless of assembling variables.

Actually, the seismic response of CLT structures is mostly dependent on the building geometry (e.g., slenderness), number, type, arrangement and design of joints used to assemble timber panels and the capability of connections to guarantee a suitable amount of plastic work. Such dependency was demonstrated by experimental tests of different shear walls [3], shake-table tests of different buildings [1,2,5] and numerical and analytical simulations of buildings and wall systems with different geometries [7,8,9]. The authors in [7,8,9] proved that slender and highly-jointed buildings can show higher displacement and dissipative capacity than squat and scarcely-jointed buildings. This is obtained by assuring a rocking behavior of each panel and by using ductile fasteners. In [7,8,9], the authors obtained different behavior factor values, depending on building geometry and panel arrangement.

The seismic performance of CLT buildings is mainly related to the capability of connections to perform plastic work, since timber elements have limited capability to deform inelastically [10,11]. Nowadays, the use of hold-down and angle bracket connections, which were originally developed for platform-frame constructions, has been extended also to CLT buildings. Nevertheless, the dissipative capacity of light-frame buildings is mainly diffused in nailing between frames and panels, *i.e.*, in the shear deformation of the wall [12,13,14,15,16,17]. This condition could be also achieved by assembling massive timber elements using ductile fasteners [18]. Contrariwise, in CLT walls, the dissipative contribution is exclusively assured by ductile connections at the base of the panels or by slender fasteners at vertical joints, being cross-wise layers reciprocally glued.

Actually, hold-downs, angle brackets and vertical joints subjected to cyclic loading show a marked pinching behavior due to the wood embedment phenomenon, which reduces the energy dissipation capability of connections. Moreover, these elements are optimized and certified for uniaxial loading (*i.e.*, only tension or only shear), while they might show undesired brittle behavior if subjected to combined loading. 

Brittle failures can occur also when the capacity design approach [19,20] is not correctly applied. The application of the capacity concept to the design of hold-downs and angle brackets is not an easy task dealing with timber structures, due to the difficulty of assuring the overstrength of brittle components *versus* ductile ones. This is because the strength of fasteners embedded in timber members [1] could be far greater than the design characteristic value evaluated according to Johansen’s theory [21], as proposed in Eurocode 5 [22]. In fact, scattering of material strength and correspondent deviation values are sensibly greater on the timber side than on the steel side of connections. Consequently, the actual strength of nails or screws might exceed the maximum strength of connected perforated steel plates, leading to unexpected fragile behavior. Therefore, when using CLT constructions, there is the need to shift the weakest element of the capacity design chain, toward the steel ductile components of the connection, the yielding load of which can be forecasted more reliably than for fasteners embedded in timber members.

The aim of this work is to demonstrate how the adoption of innovative dissipative connections, specifically developed for CLT buildings, improves the intrinsic ductility and the cyclic behavior of CLT wall systems. Various connections have already been suggested [23,24,25,26] with the intention of improving the ductile seismic response of CLT systems. A newly-developed connection element is proposed and assessed, and its main advantages are discussed. In the first section, the design phase of the connection element is illustrated. Then, its mechanical behavior is validated by means of experimental tests compared to numerical predictions. Finally, results from numerical simulations of quasi-static cyclic-loading tests are used to demonstrate the increased seismic performances of CLT shear walls anchored to foundations with the proposed connections.

## 2. Designing Process

### 2.1. Design Criteria

**Figure 1:**
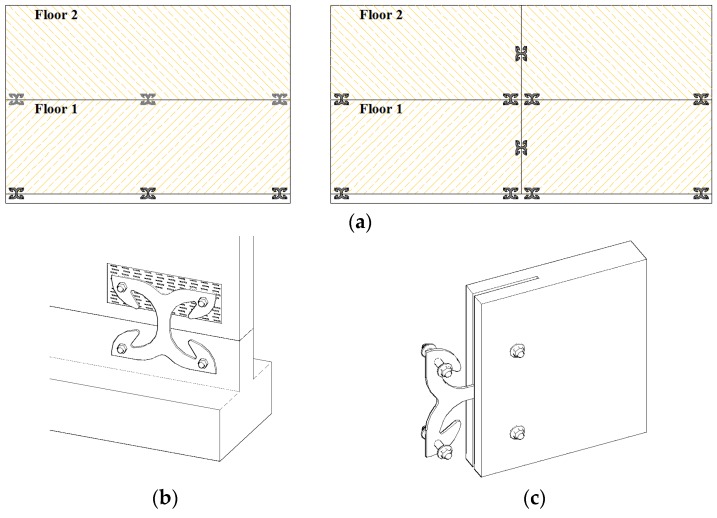
(**a**) Positioning of X-brackets in shear walls realized with CLT panels; (**b**) panel-to-foundation joint; (**c**) panel-to-panel joint.

Currently-adopted connectors for CLT panels are differentiated to prevent either sliding (angle brackets) or rocking movements (hold-downs). Conversely, the connection proposed in this work operates properly in both circumstances and has a definite behavior when subjected to mixed axial and shear forces. It assures high ductility before failure and demonstrates negligible pinching behavior, allowing one to emphasize the dissipative capacity under cyclic loading. The utilization of the proposed connection to realize both panel-to-foundation and panel-to-panel joints of shear walls is sketched in Figure 1. The adequate seismic design of a specific building involves the decision about their number, position and dimensions in fulfilment of the capacity design criteria.

The main objectives in the parametric design of the connection’s shape were: displacement capacity not less than that of alternative typically-used connections; high ductility class according to Eurocode 8 [6]; strength comparable to the traditional connectors [27]; and optimized shape with minimum scraps’ production in the manufacturing process.

**Figure 2:**
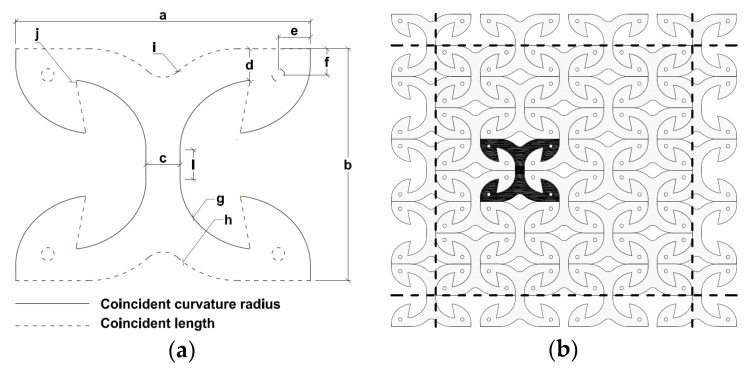
Shape and geometry of the connection: (**a**) model parameters; (**b**) manufacturing process from a steel plate.

The parametric design resulted in the original “X” shape shown in Figure 2, which can be easily and economically obtained from laser cutting of a steel sheet. Grade S275JR steel was found to be the most appropriate in order to fulfil the specified design objectives. The “X” shape connector (henceforth called “X-bracket”) is optimized to prevent localized failures and to assure diffuse yielding of material, emphasizing ductility and energy dissipation capacity. The chosen shape assures also low production costs and minimal wasting of material.

### 2.2. Parametric Design Assisted by Numerical Modelling

Once the first-tentative “X” shape is decided, a parametric Finite-Element (FE) model with modifiable geometries was used to derive the optimal dimensions, which fulfil the design criteria listed above. A 2D FE model of the X-bracket using shell elements was implemented into ANSYS Workbench [28]. The geometrical parameters chosen as the variable in the model are evidenced with letters in Figure 2a. To allow a single-cut production process, as shown in Figure 2b, reciprocal constraints among geometrical properties were imposed (see the continuous and dotted lines in Figure 2a).

An elastic-plastic constitutive law combined with a von Mises yield criterion with a kinematic hardening model was adopted to simulate steel behavior. In the parametric design phase, the elastic and hardening moduli of S275JR steel were set to 200,000 and 780 MPa, respectively, whereas yielding stress and ultimate stress were set to 275 MPa and 430 MPa, respectively. To minimize failure risk due to low-cyclic fatigue, a limit to the maximum strain of steel was imposed [29] when determining the ultimate displacement capacity for the X-brackets. Accordingly, maximum axial deformation of the X-brackets was limited between +10% and −2% (the possibility of limited compressive deformation was accounted for), while their allowable shear strain was set in the range ±6%. The non-linear geometrical analysis option was activated to account for possible buckling phenomenon under high displacements.

Numerical simulations were conducted for pure tension and pure shear cyclic loading. A total of 70 different combinations of the variable parameters led to the definition of the optimal final shape.

The parametric analysis was helpful, as modifying the length and thickness of vertical and horizontal arms allowed one to calibrate shear and tensile displacement capacity, respectively. Additionally, the variation of stiffness and strength was permitted by changing the internal curvature radius. The dimensions of the final shape listed in Table 1 allowed one to balance at the same time the strength, stiffness and ductility values of the connector and to assure similar performance, both in shear and in tension. The chosen thickness of 6.0 mm was found to be a balanced solution to withstand high loads, while avoiding premature triggering of local buckling phenomena. The internal curvature radius connecting vertical and horizontal arms was modified until the highest amount of plasticized area was involved. In particular, the high ductility in shear is mainly assured by the plastic deformation of the vertical arm, whereas in tension by the bending deformation of the horizontal arms. Results from tests and simulations described hereafter have confirmed the good balance among the main mechanical performances to assure an optimal seismic behavior of the device.

**Figure 3:**
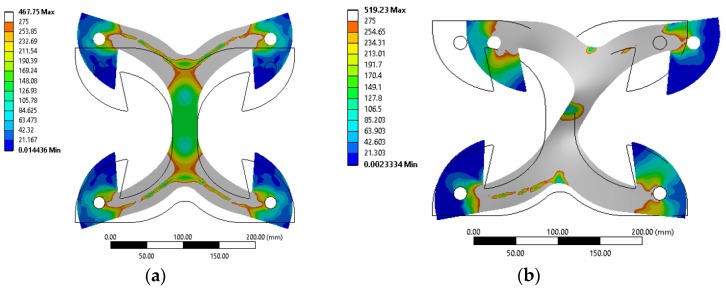
Numerical model: equivalent von Mises stress contour on deformed geometry of X-brackets: (**a**) tension loading; (**b**) shear loading. Plastic regions are evidenced in grey color.

Figure 3 shows the deformation at maximum imposed displacements, in pure tension and pure shear loading. The grey contour shows plastic regions in which the yielding stress has been exceeded. The position and extension of yielded areas vary with the loading type; however, the spread of yielding is well evident for both tests. 

### 2.3. Anchoring to the Timber Panel

In order to ensure the localization of deformations in the X-bracket, a proper design of the details of the anchorage to the timber panel is required. Fasteners used to connect X-brackets to CLT panels have to guarantee a suitable over-resistance with respect to the X-bracket itself, remaining elastic and rigid. Meanwhile, wood embedment needs to be limited. Therefore, a careful design of the anchorage details has to be applied. A possible solution is suggested in Figure 1b, where dowel-type fasteners coupled with punched metal plates are employed [30]. Adequate dimensioning of dowels and the steel punched sheet guarantees the transfer of anchoring force to the timber panel with negligible wood embedment. Alternatively, the embedment phenomenon can be reduced with the usage of toothed-plate connectors (e.g., Bulldog or Geka) or ring connectors specifically designed for these applications. Together with the sufficient overall dimensions of the X-brackets (Parameters a and b in Figure 2a), these measures allow the respecting of the adequate edge distance of the fixing devices.

## 3. Experimental Tests

After the design and optimization phases, experimental tests on prototypes have been conducted to obtain the actual cyclic behavior of X-brackets. Three tests were performed in pure tension (T1, T2, T3) and as many in pure shear (S1, S2, S3). A couple of X-brackets was placed into a rigid portal in every test. Therefore, a total of 12 equal X-brackets were tested.

Tests were performed at the Laboratory of Construction and Materials, Department of Civil, Environmental and Architectural Engineering (ICEA) of the University of Padova.

### 3.1. Test Setup and Procedure

**Figure 4:**
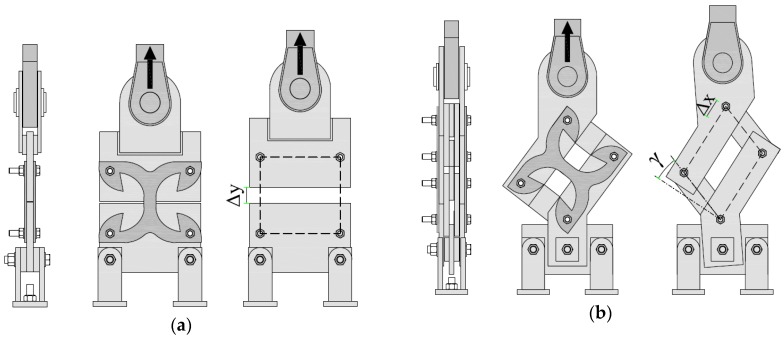
Test setup and imposed deformations. (**a**) Tension tests; (**b**) shear tests.

Two specific setups were designed for tension and shear tests (Figure 4). In order to evaluate exclusively the behavior of the X-brackets, suitable rigid steel frames were realized to transmit load from the actuators. The couple of X-brackets were fixed externally on both sides of the supporting frame without any buckling restraining elements. This arrangement allows the local buckling phenomenon for large cycles, permitting the connector to work properly, but avoids unrealistic global out-of-plane deformation. As concerns the tension configuration (see Figure 4a), the two lower fixing points were connected to a 20 mm-thick steel plate rigidly fixed to the portal. The two upper fixing points were connected to another 20 mm-thick plate fixed to the hydraulic jack through an eyebolt mechanism. The pure shear loading was obtained with an unbraced steel truss, in which the X-brackets operated as the cross-bracing element (see Figure 4b). Fifteen millimeter-thick steel plates were used for the steel truss. The whole assembly was positioned in a rotated configuration, in order to keep the loading direction as close as possible to the virtual diagonal line. In actual applications, friction might occur between the X-bracket and connected elements, so increasing the apparent strength and dissipative capacity of the connections. The unreliability of the friction effects imposes that they must be disregarded. Therefore, in all of experimental tests, polytetrafluoroethylene (Teflon-PTFE) sheets were interposed between contact surfaces to minimize friction and to determine purely the connection capacities assured by the X-bracket.

Cyclic tests were performed according to the quasi-static loading protocol recommended by EN 12512 [31]. The cyclic procedure was stopped after reaching a relative displacement of 30 mm; then the specimens were loaded monotonically until their failure. Tests were conducted under displacement control with a deformation rate of 0.02 mm/s.

### 3.2. Test Results

**Figure 5:**
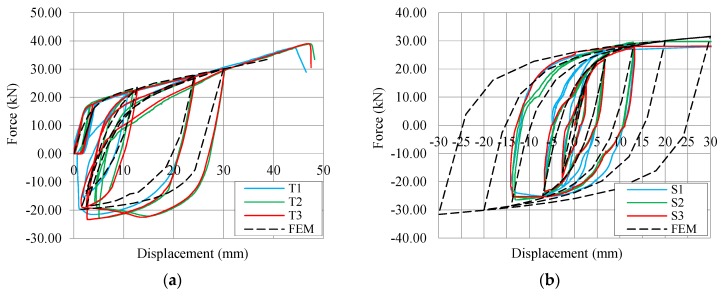
Experimental cycles in comparison to FEM results. (**a**) Tension tests; (**b**) shear tests.

Experimental tension and shear tests on X-brackets were reproduced with the same FE model adopted in the parametric design phase. Mean steel parameters introduced in the numerical models exposed later have been derived from tensile tests according to EN ISO 6892-1 [32] on specimens obtained from the same steel sheet with which the X-brackets were produced. They are specified in the following Section 4. Figure 5 plots the results of the experimental tests in comparison with those from numerical analyses.

On the 30 mm cycle of the tension test (Figure 5a), the reloading path decreased gradually due to the instability phenomenon. For the same reason, the maximum compression force measured during unloading was lower than the tension one, but still maintained a wide hysteresis area and, consequently, an appropriate dissipative capacity. The numerical model tolerably underestimated force and stiffness for the unloading sequences.

**Figure 6:**
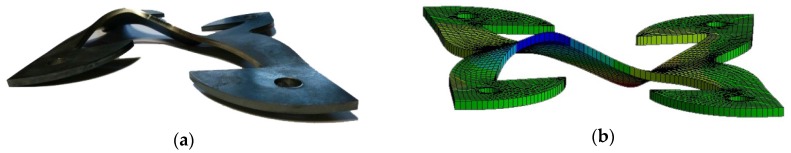
Plate buckling under shear loading. (**a**) Experimental evidence; (**b**) numerical prediction.

Results from the shear tests are plotted in terms of force-displacement curves in Figure 5b. The progressive rotation of the steel frame was accounted for the correct evaluation of the shear component of the applied force. The experimental hysteresis loops are perfectly centered on the origin of the axis, thus demonstrating the suitability of the setup configuration. The experimental cyclic shear tests were stopped at about ±15 mm due to the limitations of the test setup. Then, X-brackets were deformed monotonically up to 50, 58 and 80 mm in Tests S1, S2 and S3, respectively. In general, no noticeable strength degradation was observed in the experimental tests, and cracks induced by oligo-cyclic fatigue were not observed. Some differences appear between the experimental curves of Specimens S1, S2 and S3. They are mainly in the un-loading branches, when buckling of the web portion (clearly evidenced in Figure 6) strongly affects the response of the brackets. Perhaps, such differences are within the normal scattering of the experimental tests. 

Numerical simulations of cyclic shear tests were extended up to ±30 mm. In the range ±15 mm, the numerical results are in good agreement with the experiments, even if the numerical predictions slightly over-estimate shear force at higher displacements. This was possible as the numerical model permitted large deformations and considered also out-of-plane buckling of the X-brackets, as shown in Figure 6.

**Figure 7:**
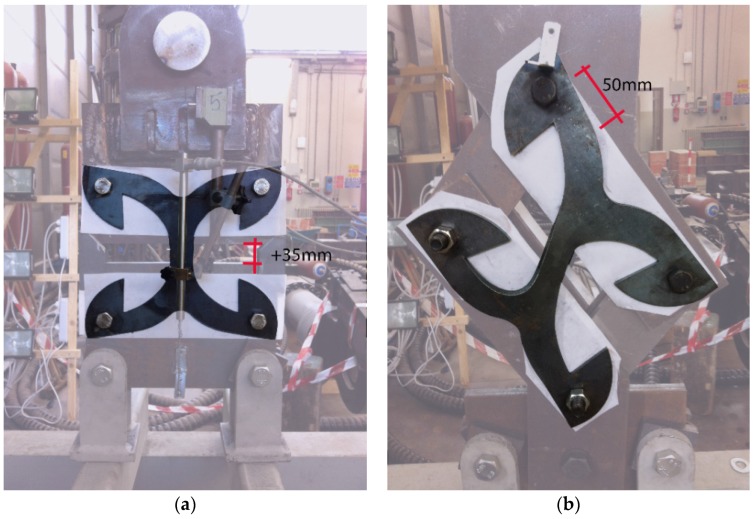
Deformed specimens: (**a**) Axial test; (**b**) shear test.

Figure 7 shows the tested specimens subjected to very large displacement (35 mm in the tension test, 50 mm in the shear test). The main evidence is that X-brackets are able to experience large plastic deformations before failure, in both loading configurations. Instability phenomena of limited parts of the specimen occurred during both shear and axial tests without impairing significantly the mechanical performance of the connectors. A direct comparison between deformed geometries in Figure 3 and Figure 7 again shows the consistency between numerical analysis and experimental validation. Specimens failed for very large displacements due to stress concentration in fillet “j” in Figure 2a. Therefore, the ductility of X-brackets could be further improved with a proper modification of this detail.

#### 3.2.1. Analysis of Test Results

The performed cyclic tests allowed one to define the main mechanical parameters for a proper characterization of the tested elements. Various methods were proposed to compute these parameters [31,33]. In this work, the envelope of the hysteresis curves was fitted using the analytical formulation proposed by Foschi and Bonac [34]. Then, Method (a) of EN 12512 [31] was chosen for both axial and shear tests, in order to obtain the best linear fitting of the envelope curve. This method is suitable to interpolate data with two well-defined linear branches, and the yielding point is defined by the intersection of these two lines. Moreover, also the equivalent elastic–plastic energy (EEEP) method [35] was used to analyze the results of the shear test, because of the almost elastic perfectly plastic behavior. From the bi-linear curves, it was then possible to obtain the elastic and post-elastic stiffness (*k_el_*, *k_pl_*), yielding point (*V_y_*, *F_y_*), failure condition (*V_u_, F_u_*) and ductility ratio *µ* and to classify the proposed connection into the appropriate ductility class, according to Eurocode 8 [6], *i.e.*, low (L), medium (M) or high (H) ductility class. Table 2 and Table 3 list the obtained results referring to a single bracket, *i.e.*, each of them represents the mean result between the couple of X-brackets contemporarily tested. Therefore, average values, standard deviations (SD) and 5% characteristic values were computed considering a sample of six elements for both tension and shear tests.

Results show that the proposed connection is characterized by a high initial stiffness and adequate strength for both tension and shear loads. However, the most valuable result is the very high value of ductility obtained, coupled with almost the null strength degradation and pinching effect.

High values of ductility are the consequences of a combination of large displacement capability *V_u_*, similar or greater than typically used connections, and an early yielding condition *V_y_*. The highest values were obtained for the axial configuration. However, ductility for the shear configuration was computed assuming *V_u_* as 50 mm, although in Test 3, failure of the specimen occurred for a displacement equal to 80 mm, whereas Tests 1 and 2 were stopped before failure. If the ultimate displacement capacity of 80 mm was assumed, ductility values in shear tests would become higher and comparable to those from axial tests. Analyzing values of initial stiffness and of yielding and ultimate forces from the two configurations, it can be observed that the connection shows a similar response when subjected to shear or axial loads.

Table 4 lists the fifth and the 95th percentile of the ultimate and yielding force (*F_0.05_* and *F_0.95_*), computed according to EN 1990 [36]. According to Fragiacomo *et al.* [20] the ratio *γ_ov_* = *F_0.95_*/*F_0.05_* is fundamental for the estimation of the overstrength factor to be used in the capacity design approach. Since only steel from a single sheet has been used for the realization of the tested X-brackets, the obtained values should be further amplified to account for the typical randomness of steel properties. Furthermore, characteristic values were obtained from a limited number of tests; therefore, the actual dispersion of the parameters may be different, resulting in slightly different overstrength values. Nevertheless, it can be stated qualitatively that the proposed connection assures limited values of *γ_ov_*, which are lower than those shown by traditional connections failing on the timber side (ductile failure). This indicates that the adoption of capacity design rules would become less onerous in designing CLT structures if the proposed connections were employed.

#### 3.2.2. Comparison with Typical Connections for CLT Walls

Values of mechanical parameters obtained for the proposed connection can be compared to analogous quantities assured by angle brackets and hold-downs typically employed in CLT buildings [27].

In comparison with a commercial hold-down having almost the same strength and stiffness, the proposed connection assures, on average, an approximately twice ultimate displacement and a ductility value about eight-times larger. In the comparison with a commercial angle bracket of similar strength, the proposed X-bracket assures, on average, twice the ultimate displacement, nine-times larger ductility value and four-times larger elastic stiffness. 

In conclusion, this novel element shows performances much higher than traditional ones, in particular when loaded in shear. This evidence testifies that this element can be properly used to improve the ductility and seismic performances of CLT buildings, even if realized with large horizontal panels and then prone to fragile sliding failure governed by shear.

## 4. Numerical Modeling of CLT Shear Walls 

**Figure 8:**
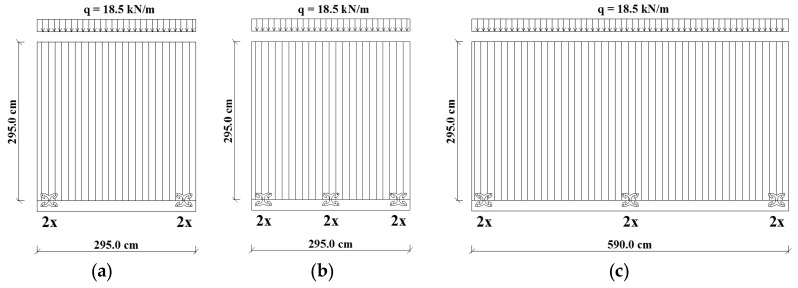
Geometry and connection arrangement of the investigated walls: (**a**) Wall A; (**b**) Wall B; (**c**) Wall C.

The FE models of three CLT shear walls (Wall A, Wall B and Wall C) were implemented into ANSYS Workbench [28]. Quasi-static cyclic-loading tests were simulated according to the EN 12512 protocol [31]. Wall A and Wall B have exactly reproduced (dimension of 2.95 m × 2.95 m, aspect ratio 1:1 and vertical distributed load 18.5 kN/m) CLT Walls I.1 and I.2, tested at Trees and Timber Institute, Italian Research Council (CNR-IVALSA) within the SOFIE (Sistema Costruttivo Fiemme) project [3]. These configurations were chosen in order to allow a direct comparison with panels representative of the CLT technology and anchored using a traditional connection system. Wall I.1 is anchored with two hold-downs and two angle brackets; Wall I.2 with two hold-downs and four angle brackets. Wall C, representing a large CLT wall without vertical joints, has dimensions equal to 5.90 m × 2.95 m (aspect ratio of 2:1) and the same vertical distributed load of the other two walls. Figure 8 shows the geometry and connection arrangement of the modeled CLT shear walls. Wall A is anchored with four X-brackets (two per side), whereas Walls B and C with six X-brackets (three per side).

### 4.1. Numerical Modelling

**Figure 9:**
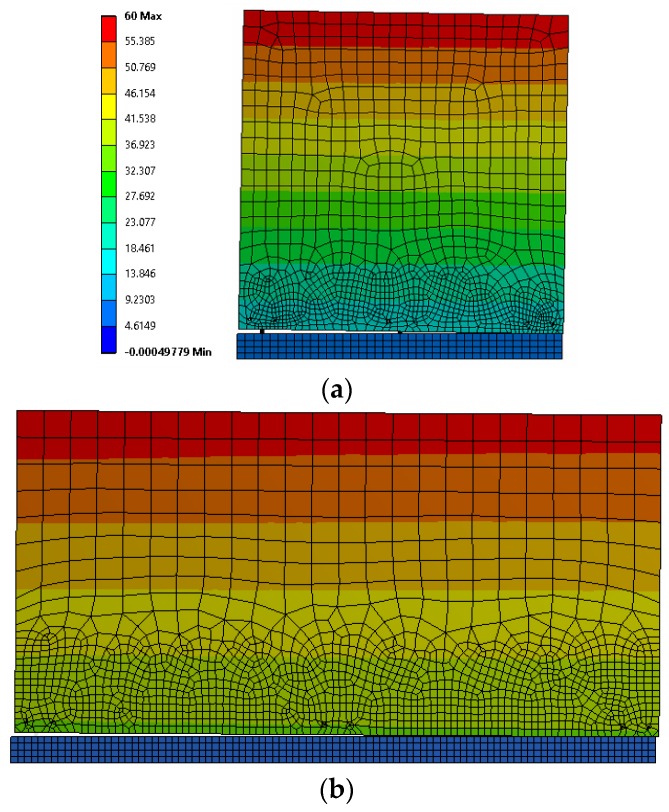
Displacement contours at the maximum imposed horizontal displacement of walls: (**a**) Wall B; (**b**) Wall C.

Linear elastic membrane elements with a thickness of 85 mm were used to simulate the timber panels (Figure 9). X-brackets were modeled with the same FE non-linear model already validated in Section 3.2, assuming that it is able to reproduce the behavior of X-brackets under combined tension and shear loads. Steel and CLT properties adopted in the numerical models are listed in Table 5. Coupling constraint equations were applied in correspondence of the fixing points to avoid relative displacements between panels and X-brackets and to permit exclusively the relative rotation (hinge connections). No gap elements were introduced at this stage to account for the possible wood embedment phenomenon. Frictionless, only compression contact elements were introduced along the interface between the wall and supporting elastic curb, so disregarding the contribution of friction effects to shear strength and dissipation. Constant distributed vertical load was distributed along the top edge, whereas imposed cyclic horizontal displacement of increasing magnitude was imposed on the middle upper point of the wall.

### 4.2. Analysis of Results

This section reports numerical results obtained for Walls A and B, compared to experimental tests on Walls I.1 and I.2, respectively [3]. Moreover, the predicted behavior of the large monolithic panel Wall C is presented.

**Figure 10:**
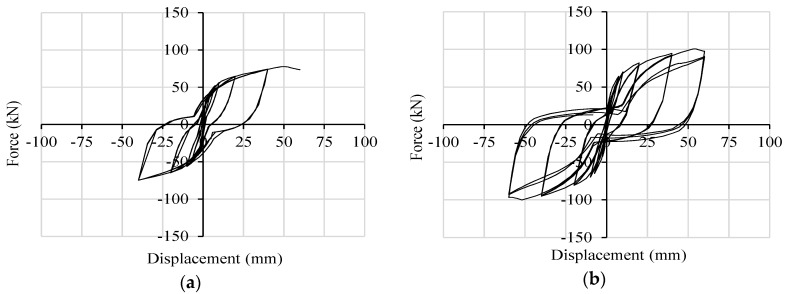
Numerical hysteresis cycles: (**a**) Wall A; (**b**) Wall B.

Figure 10 shows numerical results in terms of base shear force *vs.* displacement curves (*i.e.*, hysteresis cycles) for Wall A and Wall B. The main evidence is the different behavior of such walls in terms of strength, displacement and cycle amplitude (*i.e.*, dissipated energy capacity). Wall B (with six X-brackets) reaches higher base shear force and ultimate displacement than Wall A (with four X-brackets).

Wall A shows a good seismic response during all of the 40-mm cycles. However, the failure of the connections is obtained at an ultimate top displacement of 60 mm (drift 2%); Figure 10a. Wall B fails at 60 mm after three fully-reversed cycles with slight strength degradation due to the buckling of X-brackets, which however does not compromise the overall behavior of the wall (there is no strength degradation between the second and third cycles); Figure 10b. The two additional connectors placed in the middle, are responsible for the increased ultimate load and displacement capacities of Wall B.

**Figure 11:**
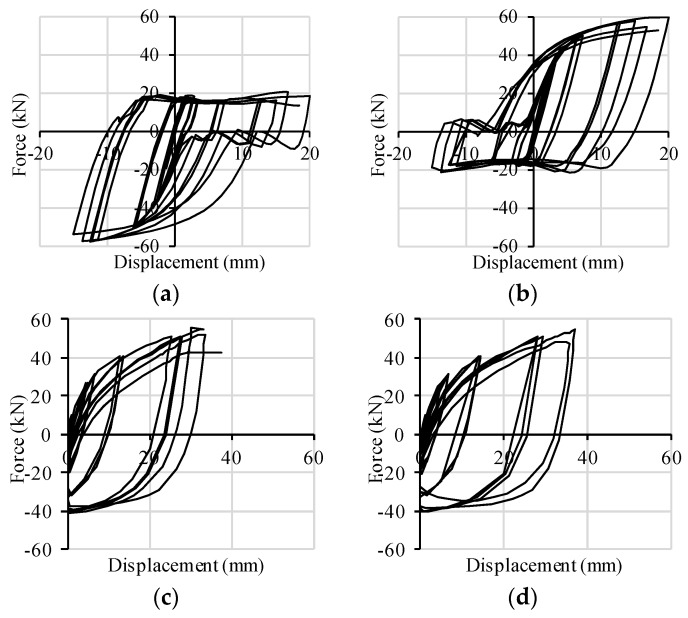
Hysteresis cycles per pair of connectors implemented in Wall A: (**a**) base slip *vs.* shear force-left corner; (**b**) base slip *vs.* shear force-right corner; (**c**) uplift *vs.* tensile force-left corner; (**d**) uplift *vs.* tensile force-right corner.

**Figure 12:**
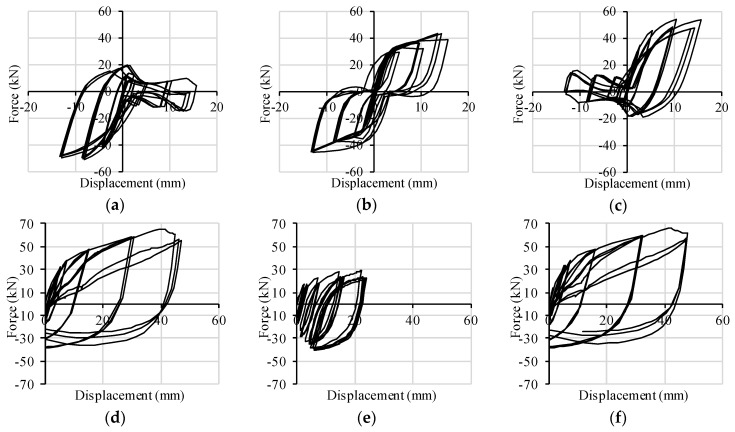
Hysteresis cycles per pair of connectors implemented in Wall B: (**a**) base slip *vs.* shear force-left corner; (**b**) base slip *vs.* shear force-middle; (**c**) base slip *vs.* shear force-right corner; (**d**) uplift *vs.* tensile force-left corner; (**e**) uplift *vs.* tensile force-middle; (**f**) uplift *vs.* tensile force-right corner.

Both of the walls fail with a combined rocking-sliding behavior, which implies a combined shear-tension loading condition in the connectors. The cyclic responses of X-brackets to such combined loading are plotted in Figure 11 for Wall A and in Figure 12 for Wall B. In the corner X-brackets, the interaction between shear and tension forces is clearly evidenced by graphs (shear capacity increases when the connection is on the compressed side of the wall). In the central brackets of Wall B, shear resistance is less weakened by contemporary traction due to rocking, and a symmetric pure shear behavior took place. Such evidence demonstrates the ability of the FE model of X-brackets to account for combined loads.

#### 4.2.1. Comparison with CLT Walls Anchored with Traditional Connections

The similitude in terms of geometry, test configuration and loading protocol between Walls A and B and Walls I.1 and I.2 tested in [3], respectively, allows a direct comparison in terms of ductility (*μ*), strength degradation (*ΔF*) and resistance (*F_y_* and *F_max_*), evaluated according to EN 12512 [31] provisions. The walls having the same number of base connections are compared (Table 6): Wall A *vs.* Wall I.1, Wall B *vs.* Wall I.2. It can be seen that the yielding and maximum resistances for the CLT walls anchored with the X-brackets are similar to those of the walls with traditional connections, whereas ductility and strength degradation are strongly improved.

The equivalent viscous damping ratio is defined as ν = *E_d_/(2π E_p_*) [31], where *E_d_* is the half-cycle dissipated energy and *E_p_* is the potential energy at the same cycle. As *E_p_* evaluation accounts for the strength degradation effect, the important contribution of the reduced pinching phenomenon might be somehow unnoticed. To assess this issue better, Table 6 lists values of the strength decrease (ΔF) and ductility (*μ*) for each group of equal amplitude cycles and also gives the viscous damping ratio (*ν*) and half-cycle dissipated energy (*E_d_*) obtained at the first and third cycle of maximum amplitude (40 mm for Walls A and I.1; 60 mm for Walls B and I.2). This comparison is consistent, as the shear strengths and displacements of the compared walls are roughly the same. The negligible strength degradation and the higher and stable energy-dissipation capacity of the walls anchored with the X-brackets lead to a marked improvement of the expected seismic response. These remarks demonstrate that the scope for which X-bracket has been developed, *i.e.*, improving the energy dissipation of shear walls without the decay of the base shear capacity and elastic stiffness, has been achieved.

#### 4.2.2. Evaluation of the Response of a Squat CLT Shear Wall

**Figure 13:**
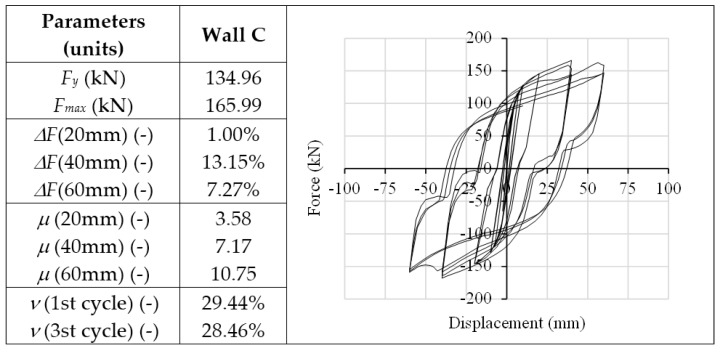
Analysis results of Wall C: plot of hysteresis cycles (right) and table with strength degradation *ΔF*, ductility *μ* and equivalent viscous damping ratio *ν*.

**Figure 14:**
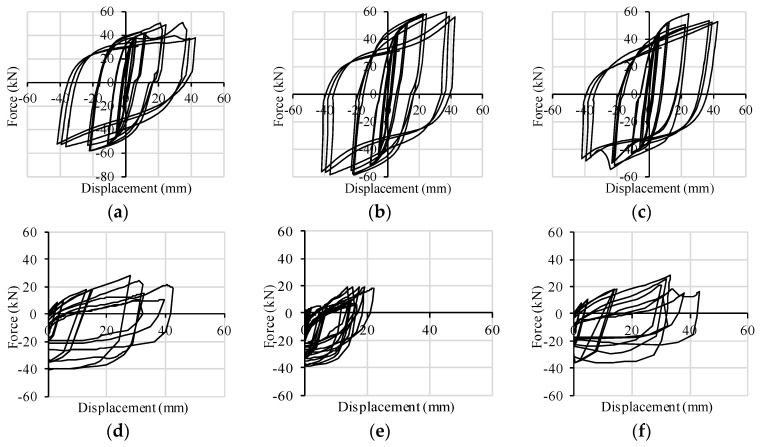
Hysteresis cycles per pair of connectors implemented in Wall C: (**a**) base slip *vs.* shear force-left corner; (**b**) base slip *vs.* shear force-middle; (**c**) base slip *vs.* shear force-right corner; (**d**) uplift *vs.* tensile force-left corner; (**e**) uplift *vs.* tensile force-middle; (**f**) uplift *vs.* tensile force-right corner.

The analysis of Wall C was performed in order to provide a comparative application test on a squat shear wall realized with a unique horizontal CLT panel. For such walls, if realized with typically-used connections, lower ductility and dissipation capacity are normally expected, since they are mainly due to the shear behavior of angle brackets, which govern the response of the entire wall in terms of force, displacement and hysteresis behavior. The use of X-brackets assures a highly dissipative seismic response of the squat wall, as reported in Figure 13. It can be seen that Wall C reaches the highest values of resistance (166.0 kN) and of viscous damping ratio *ν* (about 30.0%) of the three modeled walls. Figure 14 reports the cyclic responses of each couple of X-brackets used to anchor Wall C. With respect to Wall B, base slip prevails on up-lift. Therefore, the interaction between axial and shear forces on lateral connections is less evident. Shear strength is less impaired by traction forces due to the almost pure shear condition to which X-brackets are subjected. At the 40-mm cycles, the out-of-plane buckling of X-brackets causes slight strength degradation, but the connection still maintains its capacity, and the strength degradation is lower for higher displacements (60 mm).

## 5. Conclusions 

This paper demonstrates how the adoption of the proposed dissipative connections as a substitution of commonly-used anchoring systems allows one to improve the seismic response of CLT buildings in terms of ductility and energy dissipation capacity, even if realized with large panels and characterized by sliding behavior. The design and optimization phases of the innovative X-bracket have been described, together with the experimental validation and interpretation of the obtained results. A comparison with experimental tests of CLT shear walls with typically-used connections, characterized by similar yielding and ultimate strength, has proven that such innovative devices assure higher ductility and dissipative capacity, even if the connections undergo coupled shear-tension behavior. 

Results have confirmed also that shear walls realized with large CLT panels show a highly dissipative behavior and are classifiable into the High Ductility Class according to Eurocode 8. The augmented seismic performances of shear walls are due to the optimized shape of the steel X-bracket, which allows the connector to reach high displacement capacity, plastic deformation and diffuse yielding of material before failure.

Innovative dissipative connections should be characterized also by limited dispersion of test results, in order to make the application of the capacity design approach less onerous in designing brittle elements of a timber structure. Tests performed in this work have shown this favorable behavior for the X-bracket. However, additional tests with more specimens will be performed in order to evaluate the actual dispersion of results and consequently more precise values of overstrength. Forthcoming research will also focus on the mixed shear-tension behavior of X-brackets and the analytical definition of the strength domain, even considering different geometry ratios of X-brackets. Furthermore, experimental tests of full-scale CLT shear walls are expected.

In the seismic design of buildings, a higher behavior factor (*i.e.*, seismic force reduction factor) could be therefore allowed if X-brackets are adopted in panel-to-foundation joints and in vertical joints between panels. Furthermore, floor-to-wall connections could be designed with a suitable modification of X-bracket geometry. A capacity design approach needs to be applied in order to ensure contemporary yielding of X-brackets in each dissipative zone. An adequate assembling scheme of panels is necessary for full deployment of the ductile and dissipative capacities of X-brackets. Further studies are necessary to fully develop these concepts and for the estimation of a suitable behavior factor.

## Figures and Tables

**Table 1:** Main dimensions of X-brackets (see Figure 2a).

Parameters (Units)	Dimensions	Parameters (Units)	Dimensions
a (mm)	303.0	d (mm)	32.0
b (mm)	233.0	e (mm)	33.0
c (mm)	35.0	f (mm)	26.5

**Table 2:** Analysis of the tension test (EN 12512 method).

Parameters (units)	Test T1	Test T2	Test T3	Average	SD	5% Characteristic
*F_y_* (kN)	17.55	18.37	17.99	17.97	0.36	17.18
*V_y_* (mm)	1.89	2.01	1.98	1.96	0.06	1.83
*F_u_* (kN)	37.18	37.84	38.25	37.76	0.48	36.70
*V_u_* (mm)	44.30	47.30	47.00	46.20	1.48	42.98
*k_el_* (kN/mm)	9.31	9.12	9.08	9.17	0.11	8.94
*k_pl_* (kN/mm)	0.46	0.43	0.45	0.45	0.01	0.42
*μ*(*V_u_*) (-)	23.49	23.49	23.72	23.57	0.12	23.30
Ductility Class	H	H	H	H	-	-

**Table 3:** Analysis of the shear test (EN 12512 method and equivalent elastic–plastic energy (EEEP) method).

Parameters	Test S1		Test S2		Test S3		Average		SD		5% Characteristic	
(units)	EN	EEEP	EN	EEEP	EN	EEEP	EN	EEEP	EN	EEEP	EN	EEEP
*F_y_* (kN)	26.71	27.41	29.41	28.88	28.14	27.83	28.09	28.04	1.21	0.68	25.46	26.56
*V_y_* (mm)	2.38	2.60	4.00	4.45	4.02	4.53	3.46	3.86	0.84	0.98	1.63	1.73
*F_u_* (kN)	29.00	27.41	29.70	28.88	28.40	27.83	29.03	28.04	0.58	0.68	27.76	26.56
*V_u_* (mm)	50.00*	50.00*	58.00*	58.00*	80.00	80.00	-	-	-	-	-	-
*k_el_* (kN/mm)	11.24	10.55	7.36	6.49	7.00	6.14	8.53	7.73	2.10	2.19	3.95	2.95
*k_pl_* (kN/mm)	0.05	0.00	0.01	0.00	0.00	0.00	0.02	0.00	0.02	0.00	0.00	0.00
*μ* (*V_u_*=50mm)	21.04	19.24	12.51	11.24	12.44	11.03	15.33	13.84	4.42	4.19	5.69	4.71
Ductility Class	H	H	H	H	H	H	H	H	-	-	-	-

* Tests 1 and 2 were stopped before the ultimate displacement.

**Table 4:** Fifth and 95th percentile of the results and overstrength ratio (EN 12512 method (EN) and equivalent elastic–plastic energy (EEEP) method).

Parameters	Tension Test (EN Method)			Shear Test (EN Method)			Shear Test (EEEP Method)		
Notations (Units)	F_0.05_	F_0.95_	γ_ov_	F_0.05_	F_0.95_	γ_ov_	F_0.05_	F_0.95_	γ_ov_
F_y_ (kN)	17.18	18.76	1.09	25.46	30.71	1.21	26.56	29.52	1.11
F_u_ (kN)	36.70	38.81	1.06	27.76	30.30	1.09	26.56	29.52	1.11

**Table 5:** Main parameters used in FEM models.

Steel Connectors (Mean Values from Tensile Test According to EN ISO 6892-1:2009)		Timber Panel (Typical Mean Values for CLT Panels)	
Parameter	Value	Parameter	Value
Modulus *E* (MPa)	210,000.0	Modulus *E* (MPa)	12,500.0
Plastic Modulus *E* (MPa)	957.0	-	-
Thickness (mm)	6.0	Wall thickness (mm)	85.0
Mesh size (mm)	5.0	Mesh size (mm)	20.0/200.0
*σ_y_* (MPa)	310.0	-	-
*σ_u_* (MPa)	500.0	-	-

**Table 6:** Comparison between Wall A and Wall I.1 and between Wall B and Wall I.2.

Parameters (units)	Wall I.1	Wall A	Wall I.2	Wall B
*F_y_* (kN)	50.00	56.30	87.40	72.98
*F_max_* (kN)	70.70	77.63	104.20	100.65
*ΔF* (20mm) (-)	10.00%	0.34%	9.00%	3.10%
*ΔF* (40mm) (-)	28.50%	0.31%	14.80%	2.48%
*ΔF* (60mm) (-)	-	-	-	8.12%
*μ* (20mm) (-)	2.00	4.35	1.18	4.01
*μ* (*40mm*) (-)	4.00	8.70	2.35	8.02
*μ* (*60mm*) (-)	-	13.05	3.53	12.03
*E_d_* (1st cycle) (J)	1728	2331	3016	4748
*E_d_* (3rd cycle) (J)	1178	2174	-	3440
*ν* (1st cycle) (-)	19.60%	24.80%	16.20%	24.57%
*ν* (3st cycle) (-)	18.00%	23.90%	11.50%	20.08%
